# Effects of metabolic syndrome on aortic pulse wave velocity

**DOI:** 10.1186/s40885-016-0057-6

**Published:** 2017-01-06

**Authors:** Dong-Hyeon Lee, Ho-Joong Youn, Woo-Baek Chung, Yun-Seok Choi, Jong-Min Lee, Chul-Soo Park, Hae-Ok Jung, Hui-Kyung Jeon, Man-Young Lee

**Affiliations:** Division of Cardiology, Department of Internal Medicine, Seoul St. Mary’s Hospital, The Catholic University of Korea College of Medicine, #505 Banpo-dong, Seocho-gu, Seoul 137-701 South Korea

**Keywords:** Aorta, Pulse wave velocity, Metabolic syndrome

## Abstract

**Background:**

The purpose of this study was to compare the value and evaluate the validity of non-invasive methods for the detection of vascular stiffness in never-treated individuals with metabolic syndrome (MetS).

**Methods:**

A total of 59 subjects (mean age, 60 ± 12 years; male:female = 35:24) were enrolled in the study and were categorized into the positive MetS (MetS[+]: *N* = 32) and negative group (MetS[−]: *N* = 27), according to the parameters set by the National Cholesterol Education Program’s Adult Treatment Panel III. Pulse wave velocity (PWV) of the aorta, arm, and leg, Framingham risk score (FRS), ankle-brachial index (ABI), and carotid intima-media thickness (IMT) for vascular aging were measured for the two groups.

**Results:**

Aortic PWV (PWV*aor*) was significantly higher in MetS(+) than MetS(−) group (7.0 ± 1.4 m/s vs. 8.4 ± 1.6 m/s, *p* < 0.01), while ABI was significantly lower in MetS(+) than MetS(−) group (1.2 ± 0.1 vs. 1.1 ± 0.2, *p* = 0.03), respectively. FRS was significantly higher in MetS(+) than MetS(−) group (11 ± 5 vs. 14 ± 4, *p* = 0.05). The both mean IMT was higher in MetS(+) than MetS(−) group (right: 0.94 ± 0.20 mm vs. 0.81 ± 0.20 mm, *p* = 0.03; left: 0.93 ± 0.20 mm vs. 0.79 ± 0.20 mm, *p* = 0.03, respectively). For predicting the probability of the presence of MetS, PWV*aor* was an independent tool (*p* = 0.04; odds ratio, 1.88; 95% confidence interval, 1.03 to 3.42) and a cut-off value of PWV*aor* of 7.4 m/s showed a sensitivity of 66.7% and a specificity of 47.6%.

**Conclusions:**

We suggest that PWV*aor*, combined with traditional tools, can play an important role as a complementary or alternative tool for the detection of vascular stiffness in never-treated individuals with MetS.

## Background

Metabolic syndrome (MetS), characterized by a combination of several cardiovascular and metabolic risk factors including central obesity, dyslipidemia, elevated blood pressure, and impaired glucose tolerance [1, 2], is a worldwide health problem associated with both subclinical atherosclerosis and an increased risk of cardiovascular events [3]. It is well established that MetS not only is an accelerator of central arterial aging but also an independent predictor of cardiovascular events in older subjects [4, 5].

There are several tools for the measurement of the relationship between cardiovascular risk factors and vascular aging, including Framingham risk score (FRS) [5], which estimates 10-year risk of coronary heart disease (CHD); ankle-brachial index (ABI) [6, 7], which is used to assess and evaluate the presence of peripheral arterial disease (PAD) of the lower extremities; pulse wave velocity (PWV), pulse pressure (PP), and augmentation index (AIx), which reflect systemic arterial stiffness are significantly and independently associated with both target organ damage and increased risk for cardiovascular morbidity and mortality; subendocardial viability ratio (SEVR), which estimates myocardial perfusion relative to cardiac workload; ejection duration (ED), which reflects cardiac function by assessing the duration of left ventricular systolic ejection (systolic time interval in milliseconds) [7–9]; and intima-media thickness (IMT) of carotid artery [10–12].

The purpose of this study was to compare the value and evaluate the validity of non-invasive methods, including FRS, ABI, PWV, AIx, SEVR, ED, and carotid IMT for the detection of vascular stiffness in never-treated individuals with MetS.

## Methods

### Participants

From a total of 71 subjects, 12 subjects on anti-hypertensive drugs, oral hypoglycemic agents or insulin, lipid-lowering medication, or non-steroidal anti-inflammatory drugs (aspirin) were excluded.

Fifty-nine individuals (male [M]:female [F] ratio = 35:24; mean age, 60 ± 12 years) who visited the outpatient clinics of the St. Mary’s Hospital between March 2012 and August 2012 were enrolled in this study and they were divided into the positive MetS (Mets[+]: *N* = 32; M:F ratio = 19:13; mean age, 62 ± 12 years) and negative group (Mets[−]: *N* = 27; M:F ratio = 16:11; mean age, 59 ± 11 years), in accordance with the 2005 Adult Treatment Panel III criteria [2]. MetS(+) was defined as the presence of at least three of the following five components: (1) abdominal obesity (waist circumference >90 cm in men and >80 cm in women); (2) triglycerides ≥150 mg/dL; (3) high density lipoprotein (HDL) cholesterol <40 mg/dL in men or <50 mg/dL in women; (4) blood pressure ≥130/85 mmHg; and (5) fasting plasma glucose 100 mg/dL [2].

This study was approved by the institutional review committee of St. Mary’s Hospital. Patients were informed of the investigative nature of the study and written informed consent was obtained before enrollment (SC 14RISI0006).

### Anthropometric parameter measurement

Waist circumferences were measured using a standardized tape by the same well-trained staff. The tape was calibrated before use. The waist circumference was measured 1 inch above the umbilicus in the standing position. Systolic blood pressure (SBP), diastolic blood pressure (DBP), mean arterial pressure (MAP), and PP in both brachial artery and ankles were simultaneously measured using automated oscillometric devices (Omron HEM712C; Omron, Tokyo, Japan) with subjects in a seated position after resting quietly for 10 min. ABI was defined as the ratio between the systolic pressure measured in the ankle and that measured in the brachial artery.

### Biochemical assays

Blood samples were drawn for the analysis of levels of fasting plasma glucose, total cholesterol, triglyceride, HDL cholesterol, low density lipoprotein cholesterol and high-sensitivity C-reactive protein.

### Framingham risk score

FRS was calculated on the basis of a number of categorical variables, including age, total cholesterol level, HDL cholesterol level, SBP, treatment for hypertension, and cigarette smoking [1]. The 10-year risk for myocardial infarction and CHD is estimated from total points, and the person is categorized according to absolute 10-year risk as indicated above [1].

### Pulse wave velocity of the aorta, the arm, and the leg, augmentation index, subendocardial viability ratio, and ejection duration

PWV of the aorta, the arm and the leg (PWV*aor*, PWV*arm* and PWV*leg*), were measured using an automatic waveform analyzer (PP-1000; Hanbyul Meditech Co., Jeonju, Korea), which provides regional PWV values based on the results of electrocardiography, phonocardiography, and then automatically recorded pulse waves from four different arteries (carotid, femoral, radial, and dorsalis pedis) on the left side of the body for 10 s. PWV*aor* was determined as the velocity between the carotid and femoral arteries. PWV*arm* and PWV*leg* were calculated based on the carotid-radial and the femoral-dorsalis pedis pulse transit times, respectively.

The three main indices of cardiovascular function, namely AIx, SEVR, and ED, were measured using (GAON; Hanbyul Meditech Co., Jeonju, Korea), and were defined as follows: AIx, as the ratio of the augmentation pressure to PP; SEVR, as the ratio of the diastolic pressure time integral to the systolic pressure time integral; and ED, as the duration of systolic ejection to the total duration of a cardiac cycle. The pressures (i.e., SBP, DBP, MAP, and PP) obtained from aortic pulse wave referred to as central blood pressure.

### Carotid Intima-media thickness

Carotid IMT was measured at the common carotid and internal carotid arteries by B-mode ultrasound using a 15 MHz linear transducer (15 MHz transducer with Sonos 5500; Philips, Andover, MA, USA) following a standardized protocol. A minimum of seven measurements of common carotid far (posterior) wall were taken 20 mm proximal to the bifurcation to derive mean carotid IMT values. For statistical analysis, mean carotid IMT was calculated by averaging the thickness at four sites at the far walls of both the right and left distal common carotid arteries, two each from the right and left arteries. The physicians who performed the evaluation were blind to the health status and clinical characteristics of the study participants. A single well-trained sonographer who was blinded to clinical information made all the measurements.

### Statistical analysis

Continuous variables are expressed as mean ± standard deviation. Among five groups classified by the number of MetS components, comparisons of FRS were performed using the analysis of variance test. For comparisons between MetS(+) and MetS(−) groups, analysis for categorical data was performed using chi-square test and comparison for variable parameters was analyzed using independent *t*-test; all analyses were conducted using SAS statistical software ver. 9.1 (SAS Institute Inc., Cary, NC, USA). Multivariate logistic regression analysis was used to determine an independent tool for predicting the probability of the presence of MetS. Statistical significance was set at *p* < 0.05.

## Results

Fifty-nine individuals (M:F ratio = 35:24; mean age, 60 ± 12 years) were divided into the MetS(+) (*N* = 32; M:F ratio = 19:13; mean age, 62 ± 12 years) and MetS(−) group (*N* = 27; M:F ratio = 16:11; mean age, 59 ± 11 years). The baseline clinical and laboratory findings of the two groups are summarized in Table 1.

**Table 1:** Baseline characteristics

Variable	MetS(−) group (*N* = 27)	MetS(+) group (*N* = 32)	*p*-value
Age (yr)	59 ± 11	62 ± 12	0.20
Gender (male)	16 (59.3)	19 (59.4)	0.99
Waist circumference (cm)			
Male	88 ± 7	93 ± 7	0.07
Female	88 ± 14	88 ± 7	0.96
SBP (mmHg)	124 ± 20	128 ± 14	0.42
DBP (mmHg)	75 ± 7	74 ± 12	0.65
MAP (mmHg)	91 ± 12	93 ± 8	0.37
PP (mmHg)	52 ± 21	53 ± 11	0.85
Central SBP (mmHg)	114 ± 12	117 ± 11	0.47
Central DBP (mmHg)	78 ± 11	77 ± 7	0.73
Central MAP (mmHg)	93 ± 10	94 ± 8	0.77
Central PP (mmHg)	37 ± 14	40 ± 9	0.34
Ankle-brachial index	1.2 ± 0.1	1.1 ± 0.2	0.03
Fasting plasma glucose (mg/dL)	100 ± 8	120 ± 23	<0.01
Hemoglobin A1c (%)	5.8 ± 0.5	6.4 ± 1.3	0.45
Total cholesterol (mg/dL)	179 ± 38	182 ± 44	0.76
Triglyceride (mg/dL)	103 ± 37	184 ± 113	<0.01
High density lipoprotein cholesterol (mg/dL)	49 ± 13	42 ± 11	0.02
Low density lipoprotein cholesterol (mg/dL)	111 ± 34	108 ± 31	0.78
C-reactive protein (mg/L)	1.25 ± 2.09	2.15 ± 2.07	0.06
Framingham risk score	11 ± 5	14 ± 4	<0.01
Pulse wave velocity			
Aorta (m/sec)	7.0 ± 1.4	8.4 ± 1.6	<0.01
Arm (m/sec)	8.0 ± 1.5	8.8 ± 1.2	0.07
Leg (m/sec)	9.4 ± 1.4	9.6 ± 1.6	0.68
Augmentation index (%)	78 ± 20	89 ± 25	0.12
Subendocardial viability ratio (%)	122 ± 21	126 ± 30	0.64
Ejection duration (ms)	352 ± 43	337 ± 43	0.30
Right mean IMT (mm)	0.81 ± 0.20	0.94 ± 0.20	0.03
Left mean IMT (mm)	0.79 ± 0.20	0.93 ± 0.20	0.03

Values are presented as mean ± standard deviation or number (%)

*MetS(+)* positive metabolic syndrome, *MetS(−)* negative metabolic syndrome, *SBP* systolic blood pressure, *DBP* diastolic blood pressure, *MAP* mean arterial pressure, *PP* pulse pressure, *IMT* intima-media thickness

**Fig. 1:**
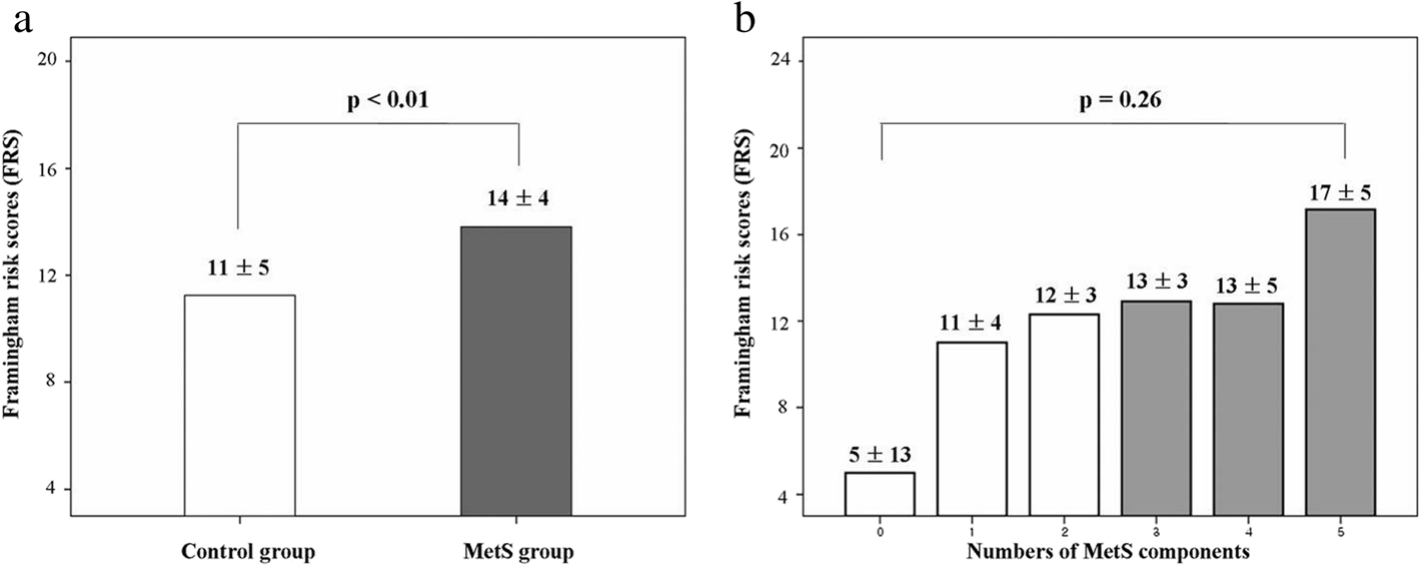
**a** Differences in FRS between MetS(+) and MetS(−) group and (**b**) among five groups classified by the number of MetS components. FRS, Framingham risk score; MetS, metabolic syndrome

**Fig. 2:**
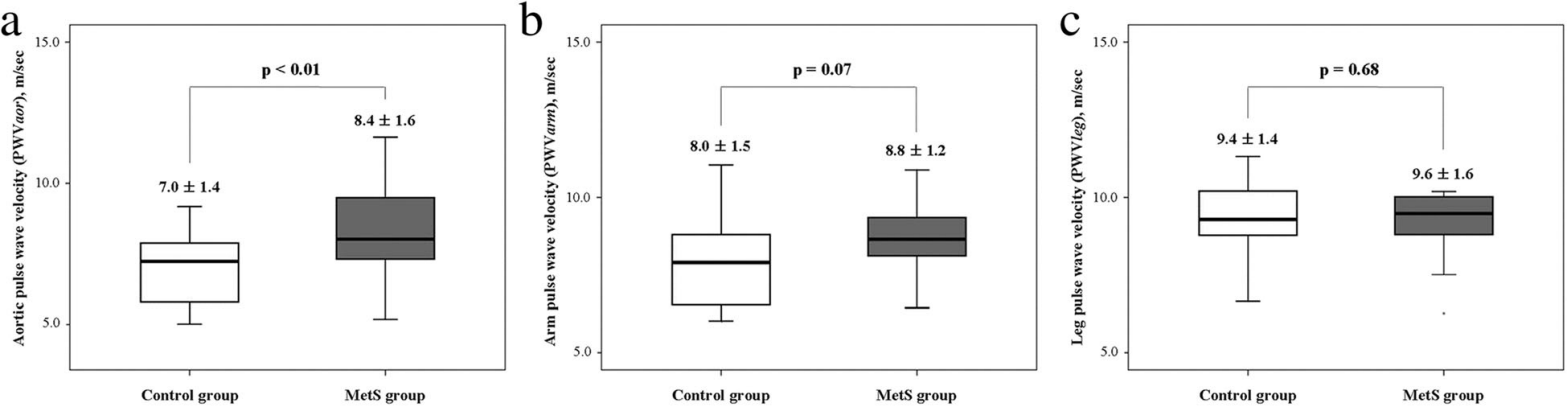
Difference in PWV of the **a** aorta, **b** arm, and **c** leg between the MetS and control group. PWV, pulse wave velocity; MetS, metabolic syndrome; PWV*aor*, pulse wave velocity of the aorta; PWV*arm*, pulse wave velocity of the arm; PWV*leg*, pulse wave velocity of the leg

FRS was significantly higher in the MetS(+) than MetS(−) group (11 ± 5 vs. 14 ± 4, *p* < 0.01) (Fig. 1a). There was no significant difference in FRS among five groups classified by the number of MetS components (0, 5 ± 13; 1, 11 ± 4; 2, 12 ± 3; 3, 13 ± 3; 4, 13 ± 5; 5, 17 ± 5; *p* = 0.26) (Fig. 1b). The ABI was significantly lower in the MetS group than in the control group (1.2 ± 0.1 vs. 1.1 ± 0.2, *p* = 0.03) and the PWV*aor* was significantly higher in the MetS(+) than MetS(−) group (7.0 ± 1.4 m/s vs. 8.4 ± 1.6 m/s, *p* < 0.01) (Fig. 2a). There was no significant difference between the control and MetS group in PWV*arm* (8.0 ± 1.5 m/s vs. 8.8 ± 1.2 m/s, *p* = 0.07) (Fig. 2b) and PWV*leg* (9.4 ± 1.4 m/s vs. 9.6 ± 1.6 m/s, *p* = 0.68) (Fig. 2c), respectively. The right and left mean IMT was higher in the MetS(+) than MetS(−) group (right mean IMT: 0.94 ± 0.20 mm vs. 0.81 ± 0.20 mm, *p* = 0.03; left mean IMT: 0.93 ± 0.20 mm vs. 0.79 ± 0.20 mm, *p* = 0.03, respectively).

**Fig. 3:**
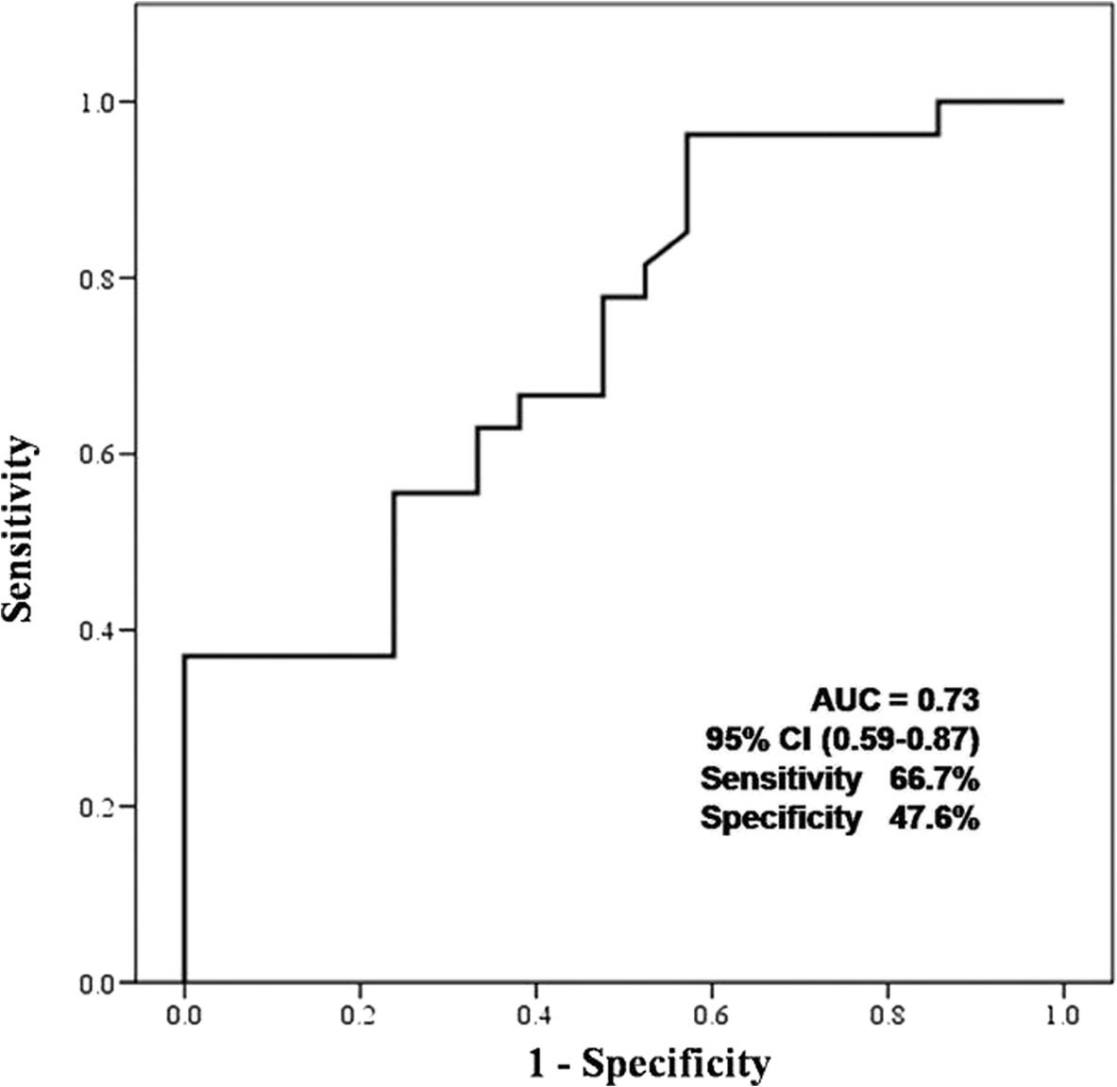
Receiver operating characteristic curve. A cut-off value of pulse wave velocity of the aorta of 7.4 m/s shows a sensitivity of 66.7% and a specificity of 47.6% for predicting the probability of the presence of metabolic syndrome. AUU, area under curve; CI, confidence interval

PWV*aor* positively correlated with FRS (*r* = 0.31, *p* = 0.03) and PWV*arm* (*r* = 0.67, *p* < 0.01), respectively (Table 2). Among the non-invasive tools, PWV*aor* was an independent tool for predicting the probability of the presence of MetS (*p* = 0.04; odds ratio, 1.88; 95% confidence interval, 1.03 to 3.42) (Table 3). A cut-off value of PWV*aor* of 7.4 m/s showed a sensitivity of 66.7% and a specificity of 47.6% for predicting the probability of the presence of MetS (Fig. 3).

**Table 2:** The relationships among the non-invasive methods for the detection of vascular aging

Variable	FRS		PWV*aor*		Right mean IMT	
	Coefficient	*p*-value	Coefficient	*p*-value	Coefficient	*p*-value
FRS	-	-	0.31	0.03	0.44	<0.01
Ankle-brachial index	0.01	0.95	0.27	0.07	0.13	0.37
PWV*aor* (m/sec)	0.31	0.03	-	-	0.27	0.11
PWV*arm* (m/sec)	0.08	0.61	0.67	<0.01	0.11	0.45
PWV*leg* (m/sec)	0.14	0.36	0.18	0.22	0.08	0.60
Augmentation index (%)	0.23	0.14	−0.08	0.61	0.32	0.04
Subendocardial viability ratio (%)	−0.02	0.91	−0.11	0.48	0.17	0.28
Ejection duration (ms)	−0.15	0.34	0.13	0.43	−0.08	0.64
Right mean IMT (mm)	0.44	<0.01	0.24	0.11	-	-
Left mean IMT (mm)	0.45	<0.01	0.26	0.09	0.71	<0.01

*FRS* Framingham risk score, *PWVaor* pulse wave velocity of the aorta, *IMT* intima-media thickness, *PWVarm* pulse wave velocity of the arm, *PWVleg* pulse wave velocity of the leg

**Table 3:** Multivariate logistic regression analysis

Variable	Adjusted odds ratio	95% Confidence interval	*p*-value
PWV of the aorta	1.88	1.03–3.42	0.04
Ankle-brachial index	0.02	0.00–3.34	0.14
Augmentation index (%)	1.03	0.98–1.08	0.27
Subendocardial viability ratio (%)	0.99	0.96–1.03	0.69
Ejection duration (ms)	1.00	0.98–1.02	0.73
Framingham risk score	1.04	0.86–1.25	0.69
Right mean IMT	8.29	0.05–1332.23	0.42
Left mean IMT	2.08	0.02–276.36	0.77

Aortic PWV was an independent tool for predicting the probability of the presence of metabolic syndrome

*PWV* pulse wave velocity, *IMT* intima-media thickness

## Discussion

MetS is a well known accelerator of central arterial aging and it is associated with an increased risk of cardiovascular events [3–5]. The present study comparing individuals with MetS and controls showed that FRS, PWV*aor* and both carotid mean IMT were significantly higher.

MetS is a risk factor for the development of coronary artery disease (CAD) and cardiovascular events. Several studies elucidated the relationships between MetS and FRS, which estimates 10-year risk of CHD. Based on data from women with suspected myocardial ischemia, Marroquin et al. [13] suggested that MetS modifies the cardiovascular risk associated with angiographic CAD. Specifically, MetS was found to be a predictor of 4-year cardiovascular risk only when associated with significant angiographic CAD. Iribarren et al. [14] reported that the presence of MetS imparts a high risk of early-onset clinical CAD, but the prognostic information associated with the syndrome is not greater than the sum of its parts. In the present study, FRS was significantly higher in the MetS than control group. Furthermore, there was no significant difference in FRS among five groups classified by the number of MetS components, which warrant further investigation.

Although our results did not show a significant difference between the control group and the MetS group in PWV*arm* and PWV*leg*, the PWV*aor* was significantly increased in the MetS group. In some studies with respect to ABI as an indicator of the presence of PAD of the lower extremities, Vogt et al. [6] showed that the ABI associated with mortality in elderly women, and Zheng et al. [7] reported the association with clinical CHD, stroke and preclinical carotid and popliteal atherosclerosis in middle-aged adults. It is well established that the PWV*aor* as an indicator of systemic vascular aging, arterial compliance or stiffness, elastic modulus, impedance, and pulse pressure amplification, which significantly and independently associated with both target organ damage and increased risk for cardiovascular morbidity and mortality [15–17]. Recently, Roman et al. [18] reported that central aortic pressure more accurately reflects loading conditions of the left ventricular myocardium, coronary arteries, and cerebral vasculature and thereby, in theory, more strongly relates to vascular disease and outcome than does brachial pressure and suggested the use of central blood pressure as a treatment target in future trials.

Although a PWV >10 m/s has been suggested as a conservative estimate of significant alterations of aortic function according to the 2007 guidelines for the management of arterial hypertension of the European Society of Hypertension and of the European Society of Cardiology [19], our investigation revealed that, among non-invasive tools including FRS and carotid IMT, PWV*aor* was an independent tool for predicting the probability of the presence of MetS, and that a cut-off value of PWV*aor* of 7.4 m/s shows a sensitivity of 66.7% and a specificity of 47.6% for predicting the probability of the presence of MetS. Blacher et al. [20] showed that PWV >12.0 m/s was associated with higher cardiovascular morbidity and mortality than PWV <9.4 m/s in patients with end-stage renal disease. Therefore, the clinical interpretation and availability of these techniques is largely limited and additional research is needed to elucidate and explain the value of PWV.

Our results revealed that both carotid mean IMT was higher in the MetS than control group. In several studies on whether MetS predicts the progression of carotid atherosclerosis, Iglseder et al. [21] reported MetS as a stronger risk factor for early carotid atherosclerosis in women, and Ahluwalia et al. [22] documented that MetS is associated with subclinical atherosclerosis using the biomarkers. Therefore, based on the present results, we suggest that MetS can play a role in the progression of subclinical carotid atherosclerosis.

Several limitations are considered in the present study. One, subclinical atherosclerosis as a vascular aging can be divided into two categories: those which increase central arterial stiffness, and those which decrease endothelial responsiveness. Although our results are in concordance with PWV*aor* increase reflecting arterial stiffness in the MetS group, no data are provided on endothelial responsiveness to detect the initial progression of subclinical atherosclerosis as a vascular aging such as results from acetylcholine stimulation tests, flow-mediated dilation and strain gauge plethysmography for the Mets and controls group, which warrants further investigation via large population together with longer-term follow-up studies are required. Another, several studies revealed that AIx differs markedly according to the difference in the study population, such as in age, gender, health status and smoking behavior [23–26]. Moreover, the time points (diurnal variations) at which AIx measurements were obtained are not reported in the present study, therefore requiring further investigation, including SEVR, ED, PWV*arm*, PWV*leg* as well as Aix [27, 28].

## Conclusions

In conclusion, among the non-invasive tools for the detection of vascular stiffness as a vascular aging in never-treated individuals with MetS, the PWV*aor* was an independent tool for predicting the probability of the presence of MetS. We suggest that PWV*aor*, combined with traditional tools, can play an important role as a complementary or alternative tool for the detection of vascular stiffness in never-treated individuals with MetS.
